# Rubella transmission and the risk of congenital rubella syndrome in Liberia: a need to introduce rubella-containing vaccine in the routine immunization program

**DOI:** 10.1186/s12879-019-4464-7

**Published:** 2019-09-18

**Authors:** Abyot Bekele Woyessa, Mohammed Seid Ali, Tiala K. Korkpor, Roland Tuopileyi, Henry T. Kohar, John Dogba, April Baller, Julius Monday, Suleman Abdullahi, Thomas Nagbe, Gertrude Mulbah, Mohammed Kromah, Jeremy Sesay, Kwuakuan Yealue, Tolbert Nyenswah, Mesfin Zbelo Gebrekidan

**Affiliations:** 1 World Health Organization Country office for Liberia, Monrovia, Liberia; 2 Republic of Liberia Ministry of Health, Monrovia, Liberia; 3 National Public Health Institute of Liberia, Monrovia, Liberia

**Keywords:** Rubella, Measles case-based surveillance, Women of childbearing age, Epidemiology, Pre-vaccine era, Liberia, West Africa

## Abstract

**Background:**

Rubella is an RNA virus in the genus *Rubivirus* within the *Matonaviridae* family. Rubella remains a leading vaccine-preventable cause of birth defects. Most African countries including Liberia do not currently provide rubella-containing vaccine (RCV) in their immunization program. We analyzed the existing surveillance data to describe rubella cases and identify the at-risk population.

**Methods:**

We conducted a retrospective descriptive statistics on the suspected-measles case-based surveillance data that obtained from the national database. Suspected-measles cases who were negative and indeterminate for measles IgM and tested for rubella IgM were extracted from the database. We used only rubella IgM positive cases to calculate trends and percentages by person, place and time. The cumulative-percent curve was used to visually describe the age distribution of rubella cases.

**Results:**

During 2017–2018, a total of 2027 suspected-measles cases with known laboratory results were reported; of which, 1307 were tested for rubella IgM. Among tested cases, 472 (36%) were positive, 769 (59%) were negative and 66 (5%) were indeterminate for rubella IgM. Female contributed 269 (57%) of the confirmed rubella cases respectively. The median age was 7 years with an interquartile range of 5–10 years. From the total rubella cases, 6 (1%) were under 1 year, 109 (23%) were 1–4 years, 207 (44%) were 5–9 years, 87 (18%) were 10–14 years and 56 (12%) were more than or equal to 15 years. Women in their reproductive-age contributed 23 (5%) of rubella cases with 17% positivity rate. Two-thirds or 307 (65%) of the cases were reported from February to May which is dry season in Liberia.

**Conclusions:**

Our analysis revealed that rubella was widely circulating in Liberia. Majority of the cases were reported among children < 15 years. However, rubella was also reported among women of reproductive age and infants < 1 year with no report of congenital rubella syndrome (CRS). Detail investigation of rubella cases among infants of < 1 year and women of reproductive age is important to uncover CRS. Establishment of CRS surveillance and the introduction of RCV in the immunization program are crucial to prevent rubella infection and avert the risk of CRS.

## Background

Rubella is an RNA virus belongs to *Rubivirus* genus within the *Matonaviridae* family [1] . The rubella virus is a human disease with no known animal reservoir and transmits to a healthy person through air droplet shed when an infected person sneezes or coughs [2]. Rubella symptoms are usually mild, and up to 50% of infections may be clinically in-apparent [3]. An Infected person is infectious and transmits the virus to healthy people starting 7 days before to 7 days after the onset of rash [4]. Infection occurs a few weeks before conception and in early pregnancy especially during the first trimester may result in miscarriage, fetal death, or congenital deformities known as congenital rubella syndrome (CRS) [5, 6].

The likelihood of CRS is high, 90%, among infants born from mothers infected by rubella virus during their early pregnancy [7]. Globally, an estimation of 100,000 babies is born with CRS every year [8, 9]. CRS incidence rate varies from 0.1–0.2 during an endemic and 0.8–4 during an epidemic per 1000 live births [6, 10]. An infant with CRS or congenital rubella infection (CRI) sheds live rubella virus for a prolonged time [11].

Regardless of the availability of safe and effective vaccine, rubella virus remains a leading vaccine-preventable cause of birth defects, especially in developing countries [6, 12]. As of 2009, only 130 (67%) of global and 2 (4%) of African countries introduced rubella-containing vaccine (RCV) in their routine immunization programs [13, 14]. As of 2017, 84% of the World Health Organization (WHO) member countries introduced rubella-containing vaccine in their program, whereas member countries in Africa region lack largely [15]. Following the introduction of rubella vaccine in many countries, global rubella vaccination coverage increased from 26% in 2007 to 52% in 2017 [16] and reported rubella cases declined by 97%, from 670,894 cases in 2000 to 22,361 cases in 2016 [17]. Rubella cases reduction mostly documented in countries providing rubella vaccine [12, 18]. In Africa, the rubella virus is still widely circulating with limited evidence of CRS [19]. Rubella incidence decreased by 48–96% in five countries in the Africa region in the post RCV introduction period as compared to the average incidence in the years before vaccine introduction [20]. To prevent rubella and CRS all WHO member countries are expected to introduce rubella vaccine in routine immunization program and reach more than 90% coverage at the national and 80% at the district level by 2020 [21].

In Liberia, an expanded program on immunization (EPI) was first launched in 1978 in accordance with WHO recommendation to all member countries. EPI have implemented in all counties and districts of Liberia as per the WHO recommendations. Children less than 1 year, as well as women of childbearing age and pregnant women, are the target groups for routine immunization. Currently, Bacillus Calmette-Guérin (BCG), pentavalent vaccine (Tetanus, Haemophilus Influenza, Diphtheria, Pertussis and Hepatitis B), oral polio vaccine (OPV), pneumococcal conjugate vaccine, measles (MCV1), yellow fever vaccine, rota vaccine, and inactivated polio vaccine (IPV) are antigens in immunization schedule for children under 1 year while tetanus toxoid is being given to every woman of childbearing age (14–49 years) in Liberia [22]. However, a rubella-containing vaccine has not yet introduced in the routine immunization program in the country. In Liberia, the routine MCV1 coverage was 80% in 2016 and 87% in 2017 according to joint WHO and United Nations Children’s Emergency Fund (UNICEF) estimation [23].

In Liberia and in all other African countries, rubella surveillance is integrated into measles case-based surveillance system. The surveillance data is collected according to the national integrated diseases surveillance and response (IDSR) technical guideline using a standard case definition for a suspected-measles case, which is an illness with fever and maculopapular generalized rash and cough, coryza or conjunctivitis or any person in whom a clinician suspects measles [24]. Blood specimen from the first five-ten suspected measles cases collected within 28 days after the onset of rash [24]. The specimens collected by the health facility’s laboratory technicians and transported to the national laboratory by a rider for health within 72 h after collection. The blood specimens are first tested for measles-specific immunoglobulin M (IgM) antibody using enzyme-linked immunosorbent assay (ELISA) and in accordance with WHO African region guideline [25] all IgM negative and indeterminate result for measles are further tested for rubella-specific IgM antibody using ELISA technique at the national reference laboratory. In Liberia, the burden, trend and transmission pattern of rubella has not clearly described. Here, we analyzed rubella data obtained from the national measles case-based surveillance database to describe rubella transmission pattern and identify at-risk population to pinpoint and recommend preventive public health interventions.

## Methods

We obtained 2 years, 2017–2018, measles case-based surveillance data from the National Public Health Institute of Liberia (NPHIL). We extracted the suspected-measles cases with known laboratory test results from the database. For this analysis, we used only Laboratory-confirmed rubella IgM positive cases as rubella cases. Descriptive statistics such as count, percentage, median and interquartile range were used to summarize each of the socio-demographic and clinical variables included in the dataset. The cumulative percentage was used to graphically describe the age distribution of rubella cases by sex. We have further categorized rubella cases into five age groups (under 1 year, 1–4 years, 5–9 years, 10–14 years and ≥ 15 years). Rubella IgM positivity rate has calculated by dividing rubella IgM positive cases to total specimens tested for rubella IgM and expressed in 100 in each category. We calculated the annualized rubella incidence rate by dividing the average of the 2 years rubella IgM positive cases to mid-year population and expressed in 100,000. To describe the possible risk of CRS, we separately analyzed rubella among women of childbearing age (15–49 years). Quantum Geographic Information System (QGIS) was used to visually demonstrate district level geographical distribution of rubella cases.

## Results

During 2017–2018, a total of 2027 suspected-measles cases with laboratory result was extracted from the national database; among them, 1307 specimens which were primarily negative and indeterminate for Measles IgM were further tested to detect rubella-specific IgM antibody. Among those tested, 472 (36%) were positive, 769 (59%) were negative and 66 (5%) were indeterminate for rubella IgM (Table 1).

**Table 1:** Laboratory results for rubella-specific immunoglobulin M antibody testing of suspected measles cases, 2017–2018, Liberia

Variables	^a^Suspected measles cases	^b^Rubella IgM tested	Rubella IgM result			
			IgM + ^c^ (%)	IgM -	Indeterminate	^d^Positivity %
Total cases	2027	1307	472	769	66	36
Year						
2017	1191	882	353 (75)	478	51	40
2018	836	425	119 (25)	291	15	28
Sex						
Female	1050	703	269 (57)	404	30	38
Male	977	604	203 (43)	365	36	34
Age Group						
< 1 Year	81	37	6 (1)	29	2	16
1–4 Years	525	287	109 (23)	172	6	38
5–9 Years	647	460	207 (44)	221	32	45
10–14 Years	329	249	87 (18)	145	17	35
> 14 Years	420	258	56 (12)	195	7	22
NA	25	16	7 (1)	7	2	44
Women of Childbearing Age	211	133	23 (5)	106	4	17

^a^Suspected measles cases with known laboratory result, ^b^suspected measles cases which are negative and indeterminate for measles IgM and tested for Rubella IgM, ^c^Proportion of confirmed rubella cases from total rubella positive cases in each category, ^d^Rubella IgM positivity rate was calculated by dividing the number of Rubella IgM positive cases to specimen tested for rubella IgM with specific variables, *IgM* Immunoglobulin M, *NA* Not available

**Fig. 1:**
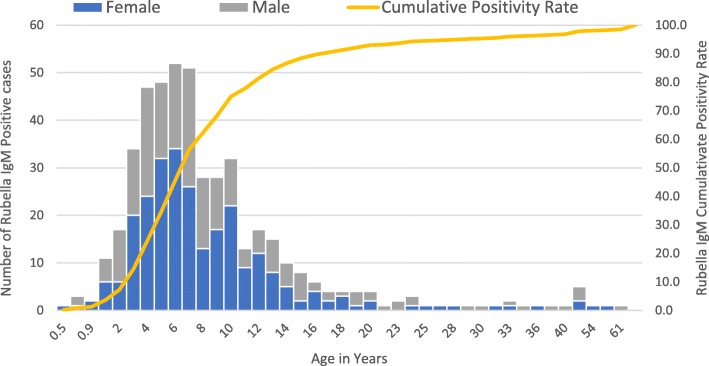
Frequency of laboratory-confirmed rubella-specific immunoglobulin M antibody testing result by age and sex with a cumulative age distribution curve, 2017–2018, Liberia, *N* = 472 (6 months to 61 years old)

Among the confirmed cases 353 (75%) were reported in 2017 and 119(25%) were reported in 2018. Female and male contributed 269 (57%) and 203 (43%) confirmed rubella cases respectively. Rubella cases have detected in all age groups with median age 7 years and interquartile ranges 5–10 years. Among the total rubella cases, 6 (1%) were under 1 year, 109 (23%) were 1–4 years, 207 (44%) were 5–9 years, 87 (18%) were 10–14 years, 56 (12%) were ≥ 15 years while the rest 7 (1%) of the cases had no age data. Women in the reproductive age group contributed 23 (5%) of the total rubella cases with a positivity rate of 17% (23/133) (Table 1 and Fig. 1).

**Fig. 2:**
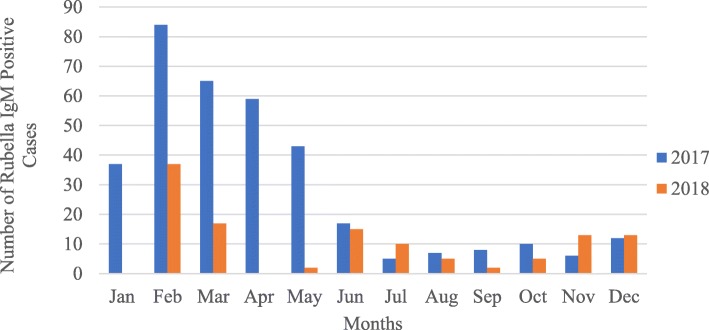
Trends of rubella IgM positive cases by month, 2017–2018, Liberia, *N* = 472 and cumulative positivity rate = 36%

From the total rubella cases, about two-thirds or 307 (65%) of them were detected from February to May. The highest cases were detected in February (26%) followed by March 82 (17%), April 59 (13%) and May 45(10%) while the lowest case was detected from July to October. Similarly, rubella IgM positivity rate was high in February 51%, April 47%, and March 39% and decreased to 15 and 17% in September and August respectively (Fig. 2).

**Fig. 3:**
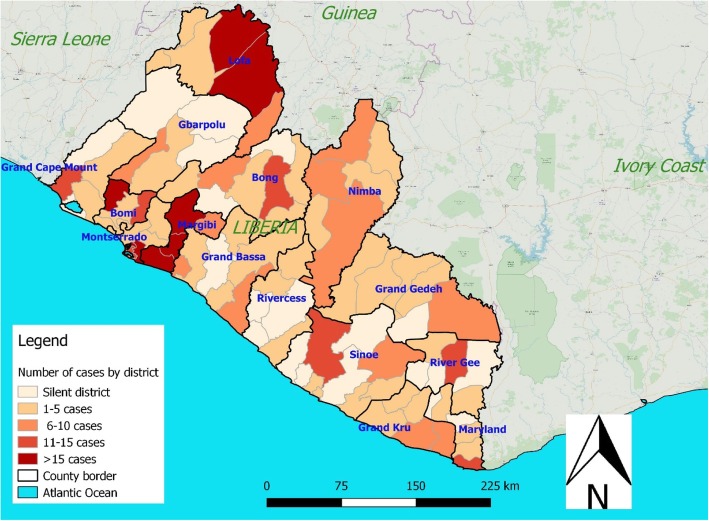
Map showing the distribution of rubella IgM positive cases and its positivity rate by district, 2017–208, Liberia

Rubella cases were reported from all counties of Liberia. Majority of the cases were reported from Montserrado 72 (15.3%), Lofa 68 (14.3%) and Margibi 66 (14.0%) while with the lowest number reported was in Rivercess 6 (1.3%). The national rubella IgM positivity rate was 36% which ranged from 14% in Rivercess County to 48% in Bomi County (Table 2 and Fig. 3). However, the rubella IgM positive incidence rate was high in Bomi followed by Grand Kru and Margibi (Table 3).

**Table 2:** Laboratory results for rubella-specific immunoglobulin M antibody testing of suspected measles cases by county, 2017–2018, Liberia

County	^a^Suspected measles cases	Rubella IgM tested	Rubella IgM result			
			IgM +	IgM -	Indeterminate	Positivity %
Montserrado	325	166	72	80	14	43
Lofa	165	152	68	77	7	45
Margibi	236	158	66	84	8	42
Bomi	92	85	41	42	2	48
Bong	163	86	33	51	2	38
Nimba	219	98	29	63	6	30
Sinoe	86	69	29	37	3	42
Grand Kru	134	82	26	55	1	32
Grand Gedeh	127	89	20	63	6	22
Maryland	119	77	20	54	3	26
Grand Bassa	118	55	19	32	4	35
Grand Cape Mount	60	42	19	22	1	45
River Gee	94	80	17	57	6	21
Gbarporlu	29	25	7	18	0	28
Rivercess	60	43	6	34	3	14
Total	2027	1307	472	769	66	36

^a^Suspected measles cases with known laboratory result, *IgM* Immunoglobulin M

**Table 3:** Annualized Rubella IgM positive cases incidence rate by county, 2017–2018, Liberia

County	Mid-Year population^a^	Rubella IgM positive cases	Annualized incidence rate per 100,000 population
Bomi	91,593	41	22.4
Grand Kru	60,152	26	4.5
Margibi	232,918	66	3.4
Sinoe	124,747	29	3.8
Lofa	313,014	68	6.2
River Gee	82,132	17	6.1
Grand Cape Mount	153,386	19	21.6
Grand Gedeh	164,360	20	10.9
Maryland	176,733	20	14.2
Bong	366,547	33	5.7
Grand Bassa	253,001	19	2.3
Gbarporlu	103,502	7	2.7
Rivercess	88,758	6	10.3
Nimba	542,289	29	3.4
Montserrado	1,550,718	72	11.6
Total	4,303,851	472	5.5

^a^Projected from the 2008 population census [26]

## Discussion

Our analysis uncovered that the rubella disease is circulating widely in Liberia and primarily infected young children under 15 years of age. The study also revealed that the rubella IgM positive cases have also detected among women of childbearing age in 5% of rubella confirmed cases with 17% positivity rate among childbearing age groups. Detection of rubella IgM positive cases among women of reproductive age suggested the possibility of rubella infection during pregnancy that could potentially lead to CRS.

The rubella positivity rate (36%) in our finding was almost similar to study conducted in Zimbabwe (37.6%) [27] and high as compared to similar other studies in the African region: for example the rubella IgM positivity rate was 30.2% in the Central Africa Republic [28], 16.6% in Côte d’Ivoire [29], 12.1–15.3% in Ethiopia [29, 30], 10.7% in Nigeria [31] and in Cameroon 9.3% [32]. The difference in positivity rate might be attributed to the nature of population settlement, topography, living conditions and study designs.

All age groups were affected while about 90% of rubella IgM positive cases were found among children under 15 years old which is also similar to other findings in Cameroon [32]. We further observed that the majority of rubella cases were reported among the younger age groups mainly 5–9 years with a median age of 7 years. This finding is similar with the study conducted on prolonged surveillance data from 40 countries in Africa in which 47% of rubella IgM positive cases detected among children 5–9 years old with an interquartile range of 4.2–7 years [19]. Other findings from similar studies demonstrated in Sub-Saharan Africa countries also supported our result [33, 34]. For instance, rubella IgM positive cases contributed 43.5% in the Central Africa Republic and 43.0% in the Democratic Republic of Congo in children 5–9 Years [28, 35] which are similar to our result. This might be because of the nature of the surveillance system. As measles surveillance is mainly focused on under 15 years children and rubella in an adult is mostly clinically inapparent, the chance of missing rubella in adult and older age group is high.

In our findings, we observed that about 5% of IgM positive rubella cases were attributed to women of childbearing age. This is supported by other similar studies in Africa. The study conducted on measles case-based surveillance data from 40 countries in Sub-Saharan Africa indicated that women of reproductive age contributed 5% of all rubella positive cases which is comparable with our finding [19]. In the Democratic Republic of Congo rubella in women of reproductive age contributed 3% of total confirmed rubella cases which are lower compared with our result. Rubella in women of childbearing age may suggest the possibility of rubella infection during pregnancy, which could potentially lead to CRS, occurs throughout the districts and remains largely undetected.

Although rubella infections were detected throughout the year, they were characterized by large peaks in the months from February to May, which corresponds with the dry and hot months in Liberia. A similar trend was observed in other countries in the tropical region, for instance, the highest number of rubella IgM positive cases were documented during the dry season in Ethiopia, Zimbabwe, and Niger [27, 30, 36, 37]. However, the reason why rubella infection increased to topmost during the dry and hot season was not clearly discussed. Some studies justified seasonal population migration from rural to urban areas was the main reason for the seasonal variation in rubella infections [38, 39]. The case in Liberia was not clear and needs further study.

Our study has some limitations as we have depended on secondary data that was primarily collected to detect measles cases. As measles surveillance is mainly focused on detection and reporting of suspected-measles among children, mild rubella cases in adult or older age groups might be potentially underreported. Furthermore, the clinical manifestation of rubella cases may not meet suspected-measles case definition as 20–50% of rubella infections do not include a rash [40] and up to 50% of infections may be in-apparent [3]. Measles surveillance is less sensitive for rubella detection [17]. We also used only suspected measles cases with known laboratory result. Because of a shortage of the reagent, a significant number of measles suspected cases were not tested for both measles and rubella IgM in 2018. The system also relies mostly on health facilities and may have missed some community cases. Hence, the data we presented here might have represented small quota of possible rubella infections in Liberia.

## Conclusions

This report provides a baseline and useful information on rubella epidemiology and transmission pattern in Liberia. Our finding demonstrated that rubella IgM positive cases had detected through the routine measles case-based surveillance system and widely circulating in Liberia. Rubella cases reported among all age groups including among infants under 1 year age and women of childbearing age. However, CRS has not detected and reported through the surveillance system. The ministry of the health of Liberia in collaboration with its partners should establish CRS surveillance according to WHO Guideline [41]. CRS sentinel surveillance needs to be established in selected health facilities mainly at maternal and child health facilities. Rubella IgM positive cases among women of childbearing age need to be further investigated and closely followed up for an outcome in case of pregnancy during infection. Similarly, rubella cases among infants in under one-year-old should be investigated for CRS. The introduction of a single dose RCV as measles-rubella (MR) in the existing routine immunization program is important to prevent rubella virus transmission and possible CRS and its public health consequence. Further study is also important to describe rubella susceptibility profile in school-aged girls and women of childbearing age in Liberia.

## Data Availability

The datasets used to prepare this report are available at the National Public Health Institute of Liberia and can be obtained from corresponding author.

## Abbreviations

BCG: Bacillus Calmette-Guérin vaccine
CRI: Congenital Rubella Illness
CRS: Congenital Rubella Syndrome
ELISA: Enzyme-Linked Immunosorbent Assay
EPI: Expanded Program on Immunization
IDSR: Integrated Diseases Surveillance and Response
IgM: Immunoglobulin M
IPV: Inactivated Polio Vaccine
NPHIL: National Public Health Institute of Liberia
OPV: Oral Polio Vaccine
QGIS: Quantum Geographic Information System
RCV: Rubella Containing Vaccine
WHO: World Health Organization
